# Inter-individual variability in mechanical pain sensation in patients with cervicogenic headache: an explorative study

**DOI:** 10.1038/s41598-022-25326-8

**Published:** 2022-11-30

**Authors:** Sarah Mingels, Wim Dankaerts, Liesbeth Bruckers, Marita Granitzer

**Affiliations:** 1 Musculoskeletal Research Unit, Department of Rehabilitation Sciences, Faculty of Kinesiology and Rehabilitation Sciences, Leuven University, 3000 Leuven, Belgium; 2 REVAL Rehabilitation Research Centre, Biomedical Research Institute, Faculty of Rehabilitation Sciences, Hasselt University, 3500 Hasselt, Belgium; 3 Interuniversity Institute for Biostatistics and Statistical Bioinformatics, Hasselt University, 3500 Hasselt, Belgium

**Keywords:** Rehabilitation, Pain

## Abstract

Currently, evidence for effective physiotherapy interventions in patients with cervicogenic headache (CeH) is inconsistent. Although inter-individual variability in pain response is predictive for successful physiotherapy interventions, it was never explored in patients with CeH. Therefore the objective of the current study was to explore inter-individual variability in mechanical pain sensation, and its association with biopsychosocial-lifestyle (BPSL) characteristics in patients with CeH. A cross-sectional explorative analysis of inter-individual variability in mechanical pain sensation in 18 participants with CeH (29–51 years) was conducted. *Inter-individual variability in mechanical pain sensation* (standard deviations (SDs), F-statistics, Measurement System Analysis) was deducted from bilateral pressure pain thresholds of the suboccipitals, erector spine, tibialis anterior. *BPSL-characteristics* depression, anxiety, stress (Depression Anxiety Stress Scale-21), quality of life (Headache Impact Test-6), sleep-quality (Pittsburgh Sleep Quality Index), and sedentary time (hours/week) were questioned. Inter-individual variability in mechanical pain sensation explained 69.2% (suboccipital left), 86.8% (suboccipital right), 94.6% (erector spine left), 93.2% (erector spine right), 91.7% (tibialis anterior left), and 82% (tibialis anterior right) of the total variability in patients with CeH. The significant p-values and large F-statistic values indicate inter-individual differences in SDs. Significant associations between (1) lower quality of life and lower SDs of the suboccipital left PPT (*p* .005), and (2) longer sedentary time and higher SDs of the suboccipital left PPT (*p* .001) were observed. Results from our explorative study could suggest inter-individual variability in mechanical pain sensation at the left suboccipitals which associates with quality of life and sedentary time. These novel findings should be considered when phenotyping patients and ‘individually’ match interventions.

## Introduction

There is consensus among clinicians and researchers on the cervical origin of cervicogenic headache (CeH)^1,2^. Interventions directed at the cervical spine are therefore recommended^3–6^. The cervical anatomical and neurophysiological basis of CeH is supported by the fundamental mechanism of convergence^7^. Potential pain generators related to CeH are innervated by C1–C3 afferent spinal nerves and include the suboccipital muscles, atlanto-occipital, atlanto-axial and C2-3 zygapophysial joints, C2-3 intervertebral disc, cervical dura mater, and vertebral arteries. Although there is no direct connection between the cervical spine and the trigeminal nerve, nociceptive information from the trigeminal and cervical regions activate neurons in the trigeminal nucleus caudalis, which extends to the C2 spinal segment and lateral cervical nucleus in the dorsolateral cervical area. This overlap between the trigeminal and cervical nerves is known as a convergence mechanism which could be responsible for pain in the face and head region^1,8^. Such mechanism also explains recurrent headaches, caused by repeated activation of cervico-occipital structures^9^.

Although the pathophysiological mechanism of CeH is well defined, non-responsiveness, low clinically relevant effect sizes^5^, and low patient-reported subjective benefit (28% reporting ≥ 50% decrease in headache frequency) of localized physiotherapy interventions targeting the cervical spine are reported^10^. It already was previously stated that not all patients with CeH were equally responsive to localized physiotherapy interventions^11,12^, and tentative attempts have been made to identify predictors of responsiveness^13^. The patient’s history and cervical physical impairments (e.g. range of motion, cranio-cervical flexion test, posture) for instance are not predicting non-responsiveness to localized physiotherapy interventions^13^. According to physiotherapists, predictors of such non-responsiveness include: previous severe trauma, neural sensitivity, immunological comorbidities, and latency of response to treatment^14^.

Pain is no longer considered to be a simple transmission of nociception, but rather an output subsequent to complex interactions between physiological, cognitive, emotional, lifestyle, and social individual inputs on neurophysiological mechanisms^15,16^. Both the subjective and personal character of experiencing pain remain major challenges for clinicians and researchers. Whereas the subjective character of pain prevents direct measurements, its personal character results from complex interactions between factors unique to that person^17,18^. Both characteristics contribute to inter- and intra-individual differences in pain reporting^16^. Inter-individual differences are unveiled by the large pain variability between individuals as a response to a standardized pain-provoking experimental stimulus^19,20^. Such differences are also observed using objective measures such as the analysis of brain morphology, cerebral activity, and neurophysiological processes during experimental pain provocation^21–23^. Thus, while the cervical origin of pain is a shared feature in patients with CeH, before defining such predictors one should realise that, variation in its experience is common^19,24^.

Individual differences in pain experience claim for an initial more patient-centred approach within study designs, rather than Randomized Controlled Trials (RCT) to evaluate the efficacy of an intervention. Although RCTs have been proven to be the gold standard to determine efficacy of interventions, they might at the beginning (i.e. understand interactions, fine-tune individual care) not be the optimal design within a heterogeneous human sample^24^. Inter-individual variability in the response to analgesic therapy has led to recommendations to design patient-centred interventions based on sound clinical reasoning^2,17^. Indeed, low success rates for the localized physiotherapeutic management of patients with CeH could relate to inter-individual variability in experiencing pain. Understanding such variability, its predictors, and unravelling its potential interactions with biopsychosocial-lifestyle (BPSL)-characteristics (e.g. gender, age, race, socio-economic status, perceived stress, trait negative affect, social support, lifestyle) seems thus crucial to explore^16,25,26^.

Therefore, as a first step we explored if in patients with CeH inter-individual differences (i.e. variability) in mechanical pain sensation can be identified, and could be linked to BPSL-characteristics. Identifying such differences and profiling the BPSL-interaction would stress the need for a more individualised patient-centred management approach. Such an intervention should include multiple modalities targeting modifiable factors that shape the individual pain experience. It is hypothesised that patients with CeH show variability in mechanical pain sensation, and that such variability is associated with BPSL-characteristics based on the BPS-model of pain^16,25^. We opted to explore variability in mechanical pain sensation since pain is often associated with mechanical hyperalgesia, i.e. decreased pressure pain threshold (PPT)^27^.

## Methods

### Design

Cross-sectional observational design to explorative variability in mechanical pain sensation within a contemporary BPSL-framework in patients with CeH.

### Sample size

A priori sample size calculation based on the Coefficient of Variation (CV) of suboccipital PPTs in patients with CeH resulted in a required sample size of 18 participants (power 80%, α = 0.05)^28^. Sample size was estimated based on the work of Van Belle et al.^29^:

$$ n = \frac{{16\left( {CV} \right)^{2} }}{{\left( {\ln \mu_{0} - \ln \mu_{1} } \right)^{2} }} $$

where CV is the coefficient of variation (CV = σ_0_/μ_0_ = σ_1_/μ_1_).

### Participants and ethics

Participants were recruited between January 2018 to August 2019 (Appendix A, Fig. A.1). The neurological staff at the headache departments of the AZ Vesalius hospitals (Tongeren and Bilzen, Belgium) identified and referred participants meeting the study’s inclusion criteria for CeH based on the international Classification for Headache Disorders, 3rd edition (ICHD)^30^. Additionally, a general call was launched at the Hasselt University, Zuyd Hogeschool, and private practice of the main researcher. Each potential participant had to be declared eligible by a neurologist (external member of the research team). The neurologist was involved in determining the inclusion and exclusion criteria, confirmed the diagnosis of CeH based on the ICHD-3 criteria^30^, and referred eligible participants with CeH to the main researcher (manual therapist with a PhD in Rehabilitation Sciences and Physiotherapy, > 10 years of clinical experience).

**Figure 1:**
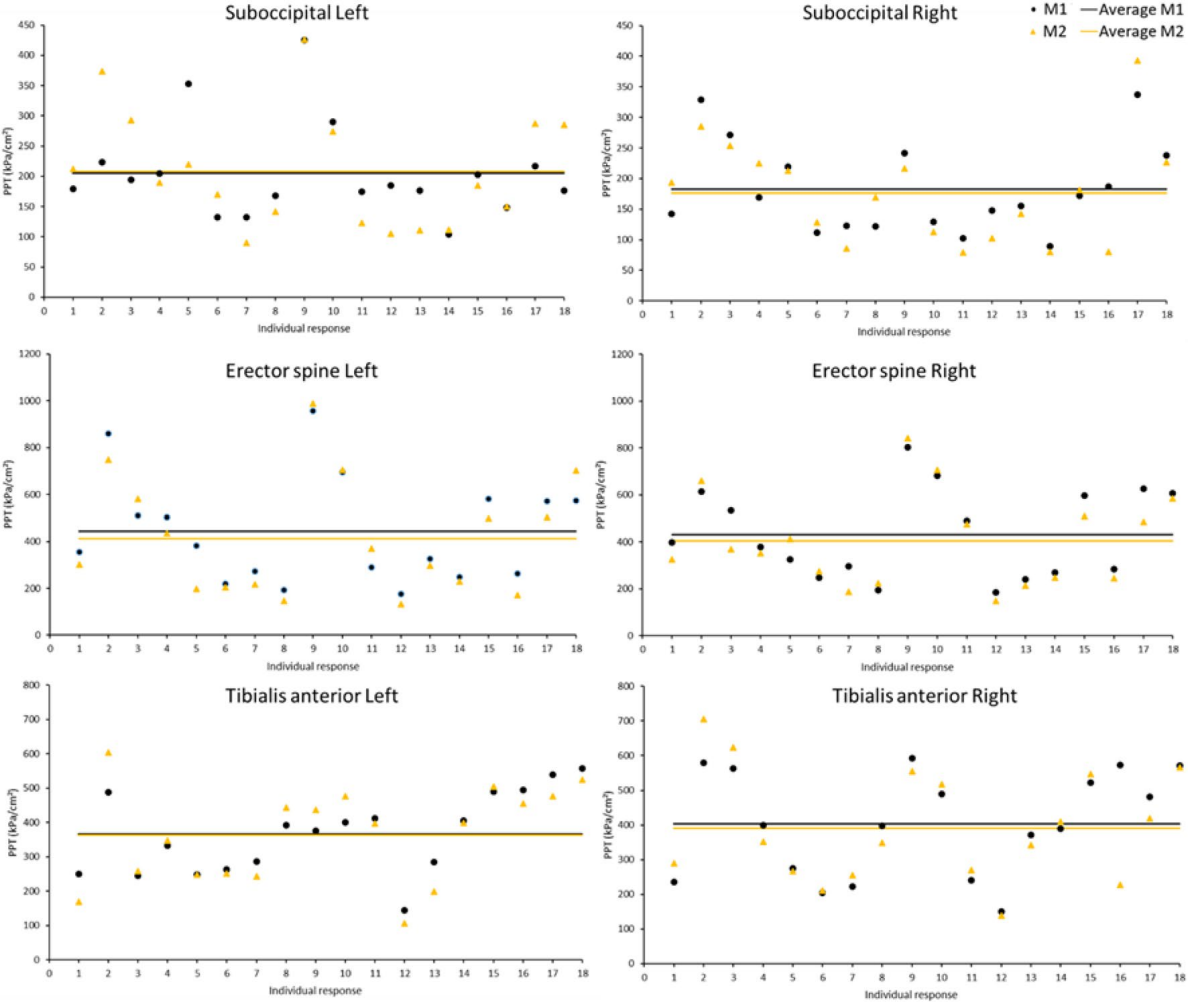
Visualisation of intra-individual (M1 vs. M2) and inter-individual (M1M2 vs. M1M2) variability, and average PPTs (full yellow and black lines) (n = 18) (PPT = Pressure Pain Threshold, M = Measurement; Full line = group average).

*Inclusion criteria* were: Dutch-speaking participants between 18 and 55 years, body mass index (BMI) between 18.5 and 24.9 kg/m^2^^31^, diagnosed with CeH according to the ICHD-3^30^ by a neurologist, normal cognitive capacity (Mini Mental State Examination test score of 30). *Exclusion criteria* were: pregnancy, physiotherapy for head- or neck-related disorders in the past month before the start of the study, serious pathology (musculoskeletal, neurological, endocrine, cardiovascular, psychiatric), medication overuse (intake of NSAID’s, opioids, acetylsalicylic acid, triptans, simple analgesics for > 10 days/month > 3 months), smoking, history of neck/head trauma, orthodontics. Nineteen participants were originally recruited and selected to compose the CeH-group. These participants were given a four-week headache-diary.

The current study was part of phase 1 of a larger project which is registered as an observational study at ClinicalTrials.gov (NCT02887638). The Medisch Ethische ToetsingsCommissie of Zuyderland and Zuyd Hogeschool (NL. 55720.09615) and the Comité Medische Ethiek of the Ziekenhuis Oost-Limburg (B371201423025) granted approval to execute the experimental protocol. Eligible participants had to read and sign the informed consent before officially being enrolled. Protection of personal data is legally determined by the Belgian law of December 8th 1992*.* All test procedures involving human participants were in accordance with the ethical standards of the institutional research committees and with the 1964 Helsinki Declaration and later amendments. The manuscript is prepared in accordance to the STrengthening the Reporting of OBservational studies in Epidemiology (STROBE) checklist.

### Outcomes, measurements, and instruments

*Primary outcome: Variability in mechanical pain sensation* (expressed via standard deviations (SDs), CV) was deducted from the PPTs (kPa/cm^2^/sec) of the bilateral suboccipitals (cephalic), erector spine at L1, and tibialis anterior (extra-cephalic). PPTs were measured with an electronic pressure algometer (Somedic AB, Stockholm, Sweden)^32–36^. PPT is defined as the minimal amount of pressure that elicits pain. Hypersensitivity over remote, extra-cephalic sites is considered a sign of central sensitization. Intrarater reliability of cervical PPT-measurements are good to excellent (ICC 0.82–0.99) in patients with headache^33,37^. Intrarater reliability of erector spine PPT-measurements are excellent (ICC 0.91 ± 0.07) in healthy participants^38^. Intrarater reliability of tibialis anterior PPT-measurements are excellent patients with neck pain (ICC 0.97)^33^. SDs were deducted from the PPT-measurements as an index for variability in mechanical pain sensation^39^.

*Secondary outcome: Headache-intensity* was measured using the 11-point Numeric Pain Rating Scale (NPRS) which ranges from 0 (no pain) to 10 (worst pain imaginable). Scores ≤ 3 correspond to mild, 4–6 to moderate, and ≥ 7 to severe pain. The meaningful clinically important change amounts to 2.5. Psychometric properties of the NPRS are solid in patients with CeH^40^.

*Secondary outcomes: BPSL-characteristics* depression, anxiety, and stress (Depression Anxiety Stress Scale 21), quality of life (Headache Impact Test 6), sleep quality (Pittsburgh Sleep Quality Index), and sedentary time (mean hours/week) were questioned through standardized Dutch questionnaires. Psychometric characteristics (e.g. validity, internal consistency, reliability) of the questionnaires used to estimate the BPSL-characteristics, and results on the completed questionnaires are described in our previous work and the Appendices (Appendix B and Appendix C, respectively)^41^.

### Test procedure

Participants were asked not to take analgesics, muscle relaxants, or caffeine-containing beverages 24 h prior to the measurements. Prophylactic treatment(s) remained unchanged^1^. Measurements (M) were performed twice (at M1 and M2) in a set-up with constant room temperature of 25 °C at the motion laboratory of Zuyd Hogeschool (Heerlen, The Netherlands)*.* A condition to be measured was a score of 0 on the 11-point NPRS for headache intensity on the test day^42,43^.

Questionnaires to estimate the BPSL-characteristics were completed first, followed by PPT- measurements of the bilateral suboccipitals, erector spine at L1 (neutral prone position), and tibialis anterior (seated with 80° knee-flexion)^38,44^. Pressure was perpendicular applied directly on the muscle belly (probe 1 cm^2^), starting at 0 to maximal 1000 kPa with a slope of 30 kPa/sec^44,45^. Participants were instructed to push the stop-button when the sensation of pressure first changed into pain. An exercise trial was performed once on the right thigh before actually measuring. PPT-measurements were executed twice (ICC 0.86–0.99) with a five-minute interval in a standardised sequential order: suboccipital left, erector spine at L1 left, tibialis anterior left, suboccipital right, erector spine at L1 right, tibialis anterior right^35,45–47^.

The entire test procedure (45 min) described above was executed and guided by the main researcher (manual therapist with a PhD in Rehabilitation Sciences and Physiotherapy, > 10 years of clinical experience).

### Statistics

Analysis was completed via JMP Pro 14 and IBM SPSS Statistics 25. Two-tailed tests at 5% level of significance were reported.

*Group characteristics.* Continuous outcomes (mean, SD) and proportions (number, %) were used to described characteristics on age, BMI, marital and socioeconomic state, and CeH.

*Mechanical pain sensation. 1. PPTs.* Continuous outcomes (mean, SD) for each muscle at M1 and M2 were compared via paired t-tests. *2. Intraclass correlation coefficients* (ICCs) were calculated to estimate intrarater reliability of the PPT-measurements. A two-way mixed-effects absolute agreement model was composed^48^. ICCs were interpreted as: values < 0.50 are indicative of poor, values between 0.50 < 0.75 of moderate, values between 0.75 < 0.90 of good, and values > 0.90 indicate excellent reliability^49^. Underlying criteria for ICC calculations (normality, homogeneity of variance) were met. *3. Variability in mechanical pain sensation*. Measurements System Analysis (% variability) and F-statistics (σ^2^Between/σ^2^Within) were used to estimate inter-individual variability. *4. Coefficients of Variation* (CV) for the PPT-measurements were calculated to express variation referred to its mean [(SD/Mean)*100].

*Associations between independent variables* age, BMI (continuous), gender, socioeconomic status, measurement (at M1 or M2) (categorical), and (1) PPTs and (2) SDs (dependent continuous) were evaluated via stepwise multiple linear regression to obtain the best model fit (i.e. smallest mean square of the error, variance inflation factor ≤ 4) (Appendix D).

*Associations between independent BPSL-variables* depression, anxiety, stress, (categorical) quality of life, sleep quality, and sedentary time (continuous), and SDs of the PPTs (dependent continuous) were evaluated via stepwise multiple linear regression to obtain the best model fit (i.e. smallest mean square of the error, variance inflation factor ≤ 4) (Appendix E). Conditions to apply linear models had to be met. SDs, to express variability of PPTs, were deducted for each participant (= i) from the PPTi,_M1_ and PPTi,_M2_, and used to build the model.

## Results

### Group characteristics

Eighteen participants with CeH met the inclusion criteria. One participant had to be excluded because of technical artefacts. Group characteristics are presented in Table 1. Appendix F provides more detailed information on the headache characteristics of the participants (Appendix F, Table F.1).

**Table 1:** Summary of the group characteristics (n = 18).

Characteristic	Result
**Age (y), mean (SD)**	40.2 (10.9)
[CI]	[34.6; 45.8]
**BMI (kg/m**^**2**^**), mean (SD)**	23.5 (3.2)
[CI]	[21.9; 25.1]
**Marital status, n (%)**	
Married	9 (50)
Living together	5 (27.8)
In a relation (not living together)	2 (11.1)
Single	2 (11.1)
**Socioeconomic status, n (%)**	
Job	
Student	2 (11.1)
Working	16 (88.9)
Services	14 (87.5)
Self-employed	2 (12.5)
Level of education	
Secondary studies	2 (11.1)
Graduate school or university	16 (88.9)
**Cervicogenic headache, n (%)**	18 (100)
Headache-duration, mean hours/episode (SD) [CI]	4.1 (1.6) [3.3; 4.9]
Headache-frequency, median days/month [IQR]	11 [10; 15.8]
Neck pain (yes), n (%)	18 (100)
Headache-intensity, mean VAS (SD) [CI]	36 (21) [26; 47]
Dominant headache-side (right), n (%)	17 (94.4)

*SD* Standard deviation, *CI* 95% Confidence Interval, *n* Number participants, *y* Years, *VAS* 100 mm visual analogue scale, *IQR* 25–75% interquartile range. Data on headache characteristics were deducted from a four-week headache-diary. Appendix F provides more detailed information on the headache characteristics of the participants (Appendix F, Table F.1).

### Mechanical pain sensation

Age, BMI, gender, level of education, employment, and the measurement (at M1 or M2) did not significantly associate with PPT-measurements, or to their SDs (Appendix D).

**Figure 2:**
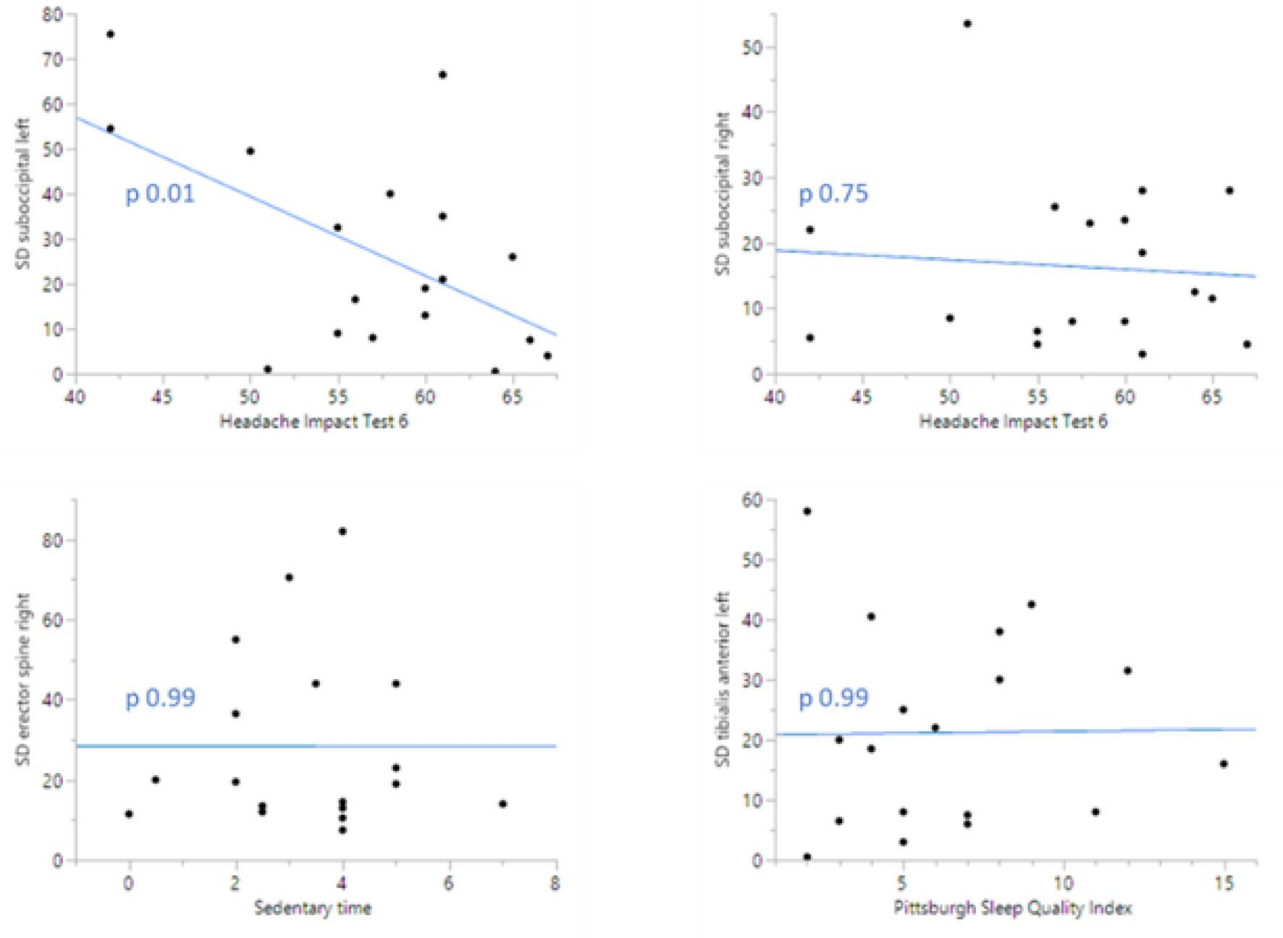
Visualisation of inter-individual differences in associations between BPSL-characteristics and SDs (SD = Standard Deviation; Sedentary time = mean hours a day/week; Full line = regression line).

- *Pressure Pain Thresholds (PPT) (Table **2**)*
  Comparison of absolute PPTs between M1 and M2 revealed no significant differences for each PPT-measurement, i.e. at the bilateral suboccipitals, erector spine, and tibialis anterior muscles.
- *Intraclass Correlation Coefficient (ICC) (Table **3**)*
  ICCs ranged from moderate (suboccipital left ICC 0.69) and good (suboccipital right ICC 0.87; tibialis anterior right ICC 0.82) to excellent (erector spine left and right ICC 0.94, 0.93, respectively; tibialis anterior left ICC 0.92).
- *Variability in mechanical pain sensation (Table **4**, Fig. **1**)*
  Inter-individual variability explained 69.2% (suboccipital left), 86.8% (suboccipital right), 94.6% (erector spine left), 93.2% (erector spine right), 91.7% (tibialis anterior left), and 82% (tibialis anterior right) of the total variability in patients with CeH. The significant *p* values and large F-statistic values indicate inter-individual differences in standard deviations.
  Averaging PPT-measurements as such at M1 and M2 smoothed out this variability. CVs ranging between 34.9 and 55.2% additionally indicate that the data have a greater level of dispersion around the mean.
- *Associations between BPSL-characteristics and variability in mechanical pain sensation (Table **5**, Appendix E)*
  Significant associations between (1) lower quality of life and smaller SDs of the suboccipital left PPT, and (2) longer sedentary time and larger SDs of the suboccipital left PPT were observed.
  No significant associations were seen between BPSL-characteristics and SDs of the remaining PPTs.
  Averaging data might mask underlying associations between BPSL-characteristics and SDs of PPTs. Individual plots of associations between BSPL-characteristics and SDs reveal inter-individual differences (Fig. 2).

**Table 2:** Summary of average PPTs (kPa/cm^2^) at M1 and M2 (n = 18).

PPT (kPa/cm^2^)	Result	*p*^a^
**Suboccipital Left**		
M1, mean (SD) [CI]	204.7 (79.4) [165.2; 244.2]	.90
M2, mean (SD) [CI]	208.3 (96.8) [160.1; 256.4]	
**Suboccipital Right**		
M1, mean (SD) [CI]	182.6 (74.9) [145.3; 219.8]	.81
M2, mean (SD) [CI]	175.9 (84.8) [133.8; 218.1]	
**Erector spine Left**		
M1, mean (SD) [CI]	443.3 (229.2) [329.3; 557.2]	.70
M2, mean (SD) [CI]	412.8 (248.7) [289.1; 536.5]	
**Erector spine Right**		
M1, mean (SD) [CI]	432.3 (189.3) [338.2; 526.5]	.66
M2, mean (SD) [CI]	403.7 (197.3) [305.6; 501.8]	
**Tibialis anterior Left**		
M1, mean (SD) [CI]	367.1 (117.5) [308.7; 425.6]	.93
M2, mean (SD) [CI]	363.4 (140.2) [293.7; 443.1]	
**Tibialis anterior Right**		
M1, mean (SD) [CI]	403.3 (150.6) [328.4; 478.2]	.82
M2, mean (SD) [CI]	391.3 (160.9) [311.3; 471.3]	

^a^Paired t-test.

*SD* Standard deviation, *CI* 95% Confidence Interval, *n* number participants.

**Table 3:** Summary of ICCs for each PPT-measurement (n = 18).

Location PPT-measurement	ICC‡
Suboccipital L	0.69
Suboccipital R	0.87
Erector spine L	0.94
Erector spine R	0.93
Tibialis anterior L	0.92
Tibialis anterior R	0.82

*L* Left, *R* Right; ‡ = deducted from two-way mixed-effects, absolute agreement models.

**Table 4:** Summary of inter-individual variability in mechanical pain sensation (n = 18).

PPT	Interindividual variability* (%)	F-ratio‡	*p*‡	CV	
Suboccipital L	69.2	5.49	**.0004**	42.1	
Suboccipital R	86.8	14.13	** < .0001**	44	
Erector spine L	94.6	36.14	** < .0001**	55.2	
Erector spine R	93.2	28.63	** < .0001**	45.7	
Tibialis anterior L	91.7	22.99	** < .0001**	34.9	
Tibialis anterior R	82	10.10	** < .0001**	38.7	

n = number participants; * = deducted from Measurements System Analysis, ‡ = deducted from the standard least square model (σ^2^Between/σ^2^Within); Bold numbers = *p* < .05; CV = Coefficient of Variation (%).

**Table 5:** Associations between BPSL-characteristics and SDs of the suboccipital left PPT (n = 18).

Variable	Estimate	Std error	Prob > |t|	Lower 95%	Upper 95%
Sedentary time	8.17	1.88	**0.0012**	4.02	12.31
HIT-6	− 1.57	0.45	**0.0053**	− 2.56	− 0.57
Anxiety [1–0]	− 8.87	6.97	0.2299	− 24.22	6.48
Anxiety [2–1]	33.96	10.43	0.0076*	11.01	56.92
Anxiety [3–2]	− 9.04	14.55	0.5474	− 41.07	22
Anxiety [4–3]	− 30.56	14.52	0.0591	− 62.51	1.39

*HIT* Headache impact test, * = no significance was observed after Tukey correction for multiple comparisons; Bold numbers = *p* < .05.

## Discussion

Although inter-individual variability in pain response is predictive for successful physiotherapy interventions, it was never explored in patients with CeH. Therefore the aim of this explorative study was to examine inter-individual variability in mechanical pain sensation, and its potential association with BPSL-characteristics in patients with CeH. Inter-individual variability in mechanical pain sensation in patients with CeH could be hypothesized based on our novel findings.

### Mechanical pain sensation varies between patients with CeH

This cross-sectional observational study is the first to explore variability in mechanical pain sensation within a contemporary BPSL-framework in patients with CeH.

Jones and O’Shaughnessy^15^ previously reported that the source of greatest variation in PPTs (ICC) in healthy participants was the inter-individual variance (70.1% of the total variance). Measurements System Analysis and F-statistics in our work demonstrate in a similar way that most variability of the PPT-measurements was due to inter-individual variability. Such variability is sometimes derogatorily referred to as error variance, i.e. a statistical term referring to sources of variance other than those of most interest to the investigator^48,49^. However, elucidating inter-individual pain variability (i.e. mechanisms and factors influencing pain experience) is of scientific and clinical significance since an enhanced understanding will provide important information to design a patient-centred model of care^50,51^. Identifying predictors influencing such variability might be of great interest since it reflects clinically realistic situations. Previous attempts to identify possible sources of variability in PPT-measurements in healthy adults revealed that male participants had 25% higher PPTs compared to female participants, anxiety levels were negatively associated with PPTs, and testing experience (i.e. higher level of self-efficacy) could contribute to greater pain tolerance^53–55^. It was further advised to reflect on the influence of psychosocial characteristics on PPT-measurements^52^.

Different ICCs were observed concerning the left (0.69) and right (0.89) suboccipitals. Regarding the left suboccipitals, eight participants endure higher pressure during M2, and regarding the right suboccipitals, five participants tolerated higher pressure during M2 (Fig. 1). This difference between left and right is also observed when comparing M1 and M2 averages (Table 2). It could be hypothesized that, at individual level, the non-provocative side for CeH (i.e. left suboccipital region) endures higher pressure due to a learning effect during M2. Maybe patients were anxious during the first measurement (PPTs at the left suboccipitals were measured before PPTs at the right suboccipitals) because the measurement took place at their ‘vulnerable’ upper-cervical spine. More pressure might have been tolerated at the left suboccipital region since this pressure could not provoke headache in 17 patients (Appendix F, Table F.1). At the provocative side for CeH (i.e. right suboccipital region), there is more consistency in the measurements, which might be explained by the peripheral provocative source being located there (17/18 patients, Appendix F, Table F.1).

Further, exploring variability in (extra)cephalic left- and right-sided PPT-measurements places previous findings, namely that patients with CeH may suffer from central sensitization, in perspective^9,41^. Although ICCs were moderate to excellent, we cannot assume that each patient with CeH encounters such central sensitization (Fig. 2). Though patients respond quite comparable to M1 and M2, PPTs fluctuate between patients. Averaging these data might give the false idea that each patient with CeH suffers from central sensitization. Consequently, therapy might be off-target.

The CVs, which ranged between 34.9 and 55.2%, indicate that our data have a greater level of dispersion around the mean. Such findings stress the need to be careful when interpreting averages^39^.

### Biopsychosocial-lifestyle characteristics associate with inter-individual variability in mechanical pain sensation

In our study we noticed that BPSL-characteristics were associated with variability in mechanical pain sensation in patients with CeH. Significant associations between (1) lower quality of life and smaller SDs of the suboccipital left PPT, and (2) longer sedentary time and larger SDs of the suboccipital left PPT were observed in patients with CeH (Table 5).

Psychosocial and lifestyle characteristics have been identified as modifiable drivers of pain and disability in other musculoskeletal pain disorders (e.g. low back pain, knee osteoarthritis)^56–59^. Therefore, we hypothesise that some patients with CeH might share a common risk profile for disability and pain, and that a shift of focus to the individual is required as suggested by Caneiro et al.^59^ for musculoskeletal pain. The variable interaction making-up a pain experience should therefore not be considered as *‘noise’*, but rather as an opportunity to develop patient-centred interventions^15,38,51^. For instance, individual differences in reporting pain at baseline were recognized as important predictors for clinical trial outcomes in patients with fibromyalgia^60^. Demographic characteristics in our study did not significantly associate with variability in mechanical pain sensation. However, it should be kept in mind that strict eligibility criteria (e.g. restricting age, BMI) were used.

Although it is generally accepted to adopt a BPSL-approach in the management of pain, a recent Delphi-study on managing patients with headache by physiotherapists proposed active mobilisation exercises, upper cervical spine mobilisations, work-related ergonomic training, and active and passive mobilisations with movement as useful in the treatment of CeH. Lifestyle advice, pain education, and cognitive therapy were not considered to be relevant^61,62^. Though, based on our new approach, and previous findings^42,43,63^, further research is needed to determine which patient is likely to benefit from which type of intervention(s) based on the patient’s BPSL-profile^61^.

### Limitations and suggestions

In the current study several statistical analyses have been used for which reflection is required. Caution is needed to generalize and interpret the results concerning the analyses of associations between BPSL-characteristics and variability in mechanical pain sensation. Several variables were selected based on a priori hypotheses and entered in the regression model, leading to many hypotheses being tested. The backward stepwise approach already downsized the model, and Bonferroni corrections were applied whenever needed^64^. The variance inflation factor (< 4) was used in case two independent variables were associated with the dependent variable. The rather small sample size (n = 18) might overestimate an effect. Post hoc power calculations for the most relevant outcomes ranged between 96 and 100%. A statistical correct interpretation (i.e. erroneously rejecting the null hypothesis) is difficult since the current study was only performed once, and no probability can be assigned to a singular, observed result. Thus, we currently have no method for deciding whether this was a false-negative or a true-negative finding and advice further research^65^. The results of this study must be interpreted in this context.

Our study investigated variability in mechanical pain sensation in adults with CeH, which of course limits generalisability of the results. Comprehensive (predictive) phenotypic profiling, including quantitative sensory testing, conditioned pain modulation, psychosocial, and lifestyle variables, is needed to evolve towards an individualized pain management program^17^ since unidimensional scales to assess pain undervalue its complexity^66^. Seventeen out of 18 patients (94.4%) reported a dominant right-sided CeH. Further analyses are needed to explore if the dominant headache-side (i.e. nociceptive information from the dominant C1-C3 level) influences these measurements. We also advice to explore if the location of PPT-measurements (i.e. close or at remote distance from the provocative source and head) associates with the consistency of such measurements.

An interesting finding in the current study was the association between quality of life and sedentary-time, and the SDs of the suboccipital left PPTs. Such association might be influenced by the ICCs of the left suboccipitals scoring the lowest. ICCs can be influenced by a number of factors including the experience of raters, learning effects, and remembering measurements from one testing occasion to the next^67^. We tried to anticipate these factors by using one experienced researcher, and a standardised protocol to measure PPTs. It could however be hypothesised that some kind of learning effect was present in the participants given that the suboccipital left PPTs were the first being measured. It could be interesting to explore to which degree the level of ICCs (i.e. poor, moderate, good, excellent) might influence associations between variability in mechanical pain sensation and BPSL-characteristics.

A condition to be measured was a score of 0 on the 11-point NPRS for headache intensity on the test day. Suffering from pain at the test day would have complicated the interpretation of the PPT-measurements, given its variation within and between individuals^51,68^. Measuring PPTs bilaterally, and at remote sites of the painful source in a pain-free period, might uncover hidden potential features of pain modulation of the individual, which are not necessarily related to a current CeH episode. We propose a prospective study design to explore if less efficient pain inhibition is secondary to the presence of pain, or if less efficient pain inhibition is primary to the clinical pain. Such studies are primordial to develop a targeted intervention for CeH.

In the current study participants were asked not to take analgesics, muscle relaxants, or caffeine-containing beverages up until 24 h prior to the measurements. Future studies, involving muscle relaxants, should apply a larger time-frame depending on the relaxant (e.g. type, dose) given the potential longer half-life of such medication.

Further, intensive longitudinal interventional designs are needed to gain more insight in inter- and intra-individual pain variability^39^ and the effect of an intervention. In particular, there is a need to evaluate both efficacy and effectiveness^69^ of an approach that assesses modifiable BPSL-factors contributing to pain using appropriate designs (e.g. single case experimental design as precursors for an RCT approach).

## Conclusion

Currently, evidence for success of physiotherapeutic managing patients with CeH is inconsistent. And, although inter-individual variability in pain response is predictive for the effect of an intervention, it was never explored in patients with CeH. Results from our explorative study could suggest inter-individual variability in mechanical pain sensation at the left suboccipitals which associates with quality of life and sedentary time.

Future studies should evaluate the efficacy of phenotyping patients in a more individualised multidimensional patient-centred intervention for CeH.

## Supplementary Information

Supplementary Information.

## Data Availability

Raw data generated during this study are included in the supplementary file (Appendix G).

## Abbreviations

BMI: Body mass index
BPS(L): Biopsychosocial lifestyle
CeH: Cervicogenic headache
CI: Confidence interval
CV: Coefficient of variation
DASS-21: Depression anxiety stress scale
ES: Effect size
HIT-6: Headache impact test 6
ICC: Intraclass correlation coefficient
ICHD: International classification of headache disorders
IQR: Inter quartile range
L: Left
M: Measurement
n: Number of participants
NPRS: Numeric pain rating scale
PPT(s): Pressure pain threshold(s)
PSQI: Pittsburgh sleep quality index
R: Right
RCT: Randomized controlled trial
SD(s): Standard deviation(s)
STROBE: Strengthening the reporting of observational studies in epidemiology
VAS: Visual analogue scale
