# Supplementary material for: Inter-individual variability in mechanical pain sensation in patients with cervicogenic headache: an explorative study
Source: Sci Rep. 2022 Nov 30;12:20635. doi: 10.1038/s41598-022-25326-8 (PMC9712535; doi:10.1038/s41598-022-25326-8)
Supplement: Supplementary file 1 — Supplementary Information. [file 41598_2022_25326_MOESM1_ESM.docx]

**Inter-individual variability in mechanical pain sensation in patients with cervicogenic headache: An explorative study**

Sarah Mingels^1,2*^, Wim Dankaerts^1^, Liesbeth Bruckers^3^, and Marita Granitzer^2^

^1^ Musculoskeletal Research Unit, Department of Rehabilitation Sciences, Faculty of Kinesiology and Rehabilitation Sciences, Leuven University, 3000, Leuven, Belgium

^2^ REVAL Rehabilitation Research Centre, Biomedical Research Institute, Faculty of Rehabilitation Sciences, Hasselt University, 3500, Hasselt, Belgium

^3^ Interuniversity Institute for Biostatistics and Statistical Bioinformatics, Hasselt University, 3500, Hasselt, Belgium

* corresponding author: [sarah.mingels@kuleuven.be](mailto:sarah.mingels@kuleuven.be)

**Appendix A – Flowchart of the recruitment and enrolment procedure**

***
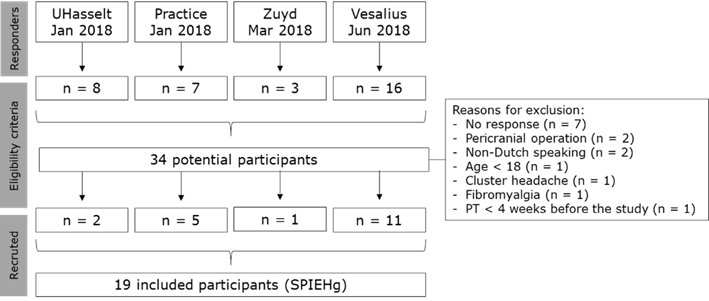
***

CeH-group

**Figure A.1.** Flowchart of recruitment (n = number responders; PT = Physiotherapy).

**Appendix B – Interpretation secondary outcomes**

Items on the ***Depression Anxiety Stress Scale-21*** are scored according a Likert-scale (0 = did not apply to me at all, and 3 = applied to me very much or most of the time). Table B.1 provides a summary of the interpretation of the scores on each subscale.

**Table B.1.** Interpretation and recommended cut-off scores for the subscales of the Depression Anxiety Stress Scale-42 [1].

|  | Depression | Anxiety | Stress |
| --- | --- | --- | --- |
| Normal | 0-9 | 0-7 | 0-14 |
| Mild | 10-13 | 8-9 | 15-18 |
| Moderate | 14-20 | 10-14 | 19-25 |
| Severe | 21-27 | 15-19 | 26-33 |
| Extremely severe | > 28 | > 20 | > 34 |

Scores the Depression Anxiety Stress Scale-21 need to be multiplied by two.

Questions on the ***Headache Impact Test-6*** are completed based on the level of agreement on a 0-4 Likert scale (never, rarely, sometimes, very often, always), and numerically converted: 6, 8, 10, 11, and 13, respectively. The impact of headache on daily life depends on the total score which varies between 36 and 78. Table B.2 provides a summary of the interpretation of the scores.

**Table B.2.** Interpretation and recommended cut-off scores for the Headache Impact Test-6 [2,3].

| Score | Interpretation |
| --- | --- |
| ≤ 49 | No to little impact of headache on daily life |
| 50-55 | Headache seems to affect daily life |
| 56-59 | Headache has a significant impact on daily life |
| ≥ 60 | Headache has a very heavy impact on daily life |

The ***Pittsburgh Sleep Quality Index*** is scored 0 (no problem) to 3 (serious problem). A total score exceeding 5/21 on the Pittsburgh Sleep Quality Index indicates poor sleep quality. Table B.3 provides a summary of the interpretation of the scores.

**Table B.3.** Interpretation and recommended cut-off scores for the lifestyle characteristics [4].

| Lifestyle characteristics | Interpretation | | |
| --- | --- | --- | --- |
| Pittsburgh Sleep Quality Index  ≤ 5  6-7  ≥ 8 | Optimal  Borderline  Poor | |  |
| Sedentary-time hours/day  0 hours  < 3 hours  3 to 6 hours  ≥ 7 hours | No  Little  Moderate  Severe |  |  |

**Appendix C – Results secondary outcomes**

Table C.1 provides a summary of the results of the secondary outcomes [5].

**Table C.1.** Summary: Psychosocial and lifestyle characteristics in patients with CeH (n = 18).

| Psychosocial characteristics | CeH-group |
| --- | --- |
| Depression, Anxiety, Stress Scale-21, n (%)  Depression  Normal (0 – 9)  Mild (10 – 13)  Moderate (14 – 20)  Severe (21 - 27)  Extreme (≥ 28)  Anxiety  Normal (0 – 7)  Mild (8 – 9)  Moderate (10 – 14)  Severe (15 – 19)  Extreme (≥ 20)  Stress  Normal (0 – 14)  Mild (15 – 18)  Moderate (19 – 25)  Severe (26 – 33)  Extreme (≥ 34) | 10 (55.6)  2 (11.1)  2 (11.1)  3 (16.7)  1 (5.6)  7 (38.9)  6 (33.3)  2 (11.1)  1 (5.6)  2 (11.1)  7 (38.9)  4 (22.2)  1 (5.6)  3 (16.7)  3 (16.7) |
| Headache Impact Test-6, n (%)  Little/none (≤ 49)  Substantiate (50 - 55)  Moderate (56 - 59)  Severe (> 60)  p^a^ within the group | 2 (11.1)  1 (5.6)  6 (33.3)  9 (50)  **.008** |

| Lifestyle characteristics | CeH-group |
| --- | --- |
| Pittsburgh Sleep Quality Index, n (%)  Optimal (≤ 5)  Borderline (6 – 7)  Poor (≥ 8) | 7 (38.9)  5 (27.8)  6 (33.3) |
| Sedentary-time: free-time hours/day, n (%)  No (0 hours)  Little (< 3 hours)  Moderate (3 – 6 hours)  Severe (≥ 7 hours) | 1 (5.6)  7 (38.9)  9 (50)  1 (5.6) |

SD = Standard Deviation; CI = 95% Confidence Interval; n = number participants; Bold numbers = p < .05.

**Appendix D –** **Associations between demographic independent variables and outcomes**

Associations between age, BMI, gender, level of education, employment, and measurement (at M1 or M2) and PPT-measurements were analysed through multiple linear regression. An overview of the statistical significance (p-values) of these effects in is provided in Table D.1.

**Table D.1.** Summary of associations between independent variables and PPT-measurements in patients with CeH (n = 18).

| Outcome | Age (p) | BMI (p) | Gender (p) | LOE (p) | Job (p) | Measurement (p) |
| --- | --- | --- | --- | --- | --- | --- |
| PPT sub L | .70 | .55 | .28 | .66 | .62 | .83 |
| PPT sub R | .46 | .41 | .25 | .84 | .72 | .51 |
| PPT ES L | .47 | .35 | .23 | .09 | .37 | .10 |
| PPT ES R | .14 | .63 | .20 | .05 | .27 | .09 |
| PPT Tib L | .08 | .92 | .41 | .23 | .29 | .77 |
| PPT Tib R | .53 | .65 | .31 | .08 | .45 | .60 |
| SD PPT Sub L | .77 | .27 | .40 | .09 | .07 | 1 |
| SD PPT Sub R | .47 | .29 | .49 | .95 | **< .0001^‡^** | 1 |
| SD PPT ES L | .12 | .79 | .18 | .22 | .06 | 1 |
| SD PPT ES R | .81 | .36 | .28 | .96 | .88 | 1 |
| SD PPT Tib L | .49 | .36 | .09 | .11 | .07 | 1 |
| SD PPT Tib R | .61 | .50 | .29 | .39 | **< .0001^‡^** | 1 |

SD = Standard Deviation; LOE = Level of Education; Job = Employment; ES = Erector Spine L1; Tib = Tibialis anterior; L = Left; R = Right; ‡ = unemployed participants (2 participants) showed significantly higher SD for the PPT suboccipital and tibialis anterior right compared to employed participants after Tukey correction for multiple comparisons.

**Appendix E – Associations between independent BPSL-variables and outcomes**

The tables below provide information on the details of the multiple regression models concerning associations between BPSL-variables and SDs in patients with CeH (n = 18). Final models were chosen based on the best model fit (i.e. smallest root square of the error, Tukey corrections for multiple comparisons).

**Suboccipital L**

**Table E.1**. Summary of significant associations between SD of the suboccipital left and BPSL-characteristics.

| **Term** | **Estimate** | **Std Error** | **t Ratio** | **Prob>\|t\|** | **Lower 95%** | **Upper 95%** |
| --- | --- | --- | --- | --- | --- | --- |
| Intercept | 90.052392 | 27.50488 | 3.27 | **0.0074** | 29.514559 | 150.59023 |
| Sedentary time | 8.1666319 | 1.882557 | 4.34 | **0.0012** | 4.023151 | 12.310113 |
| HIT-6 | -1.567401 | 0.452324 | -3.47 | **0.0053** | -2.56296 | -0.571842 |
| Anxiety[1-0] | -8.866443 | 6.974633 | -1.27 | 0.2299 | -24.21751 | 6.4846214 |
| Anxiety[2-1] | 33.961126 | 10.42926 | 3.26 | **0.0076*** | 11.006483 | 56.91577 |
| Anxiety[3-2] | -9.035519 | 14.55444 | -0.62 | 0.5474 | -41.06962 | 22.998579 |
| Anxiety[4-3] | -30.55944 | 14.51622 | -2.11 | 0.0591 | -62.50942 | 1.3905347 |

HIT = Headache Impact Test, *= no significance was observed after Tukey correction for multiple comparisons; Bold numbers = significance (p < .05).

**Table E.2**. Summary of non-significant associations between SD of the suboccipital left and BPSL-characteristics.

| **Term** | **Estimate** | **Std Error** | **t Ratio** | **Prob>\|t\|** | **Lower 95%** | **Upper 95%** |
| --- | --- | --- | --- | --- | --- | --- |
| Intercept | 36.801882 | 16.03586 | 2.29 | 0.0406 | 1.8627491 | 71.741015 |
| PSQI | -2.053772 | 3.004419 | -0.68 | 0.5072 | -8.599839 | 4.4922941 |
| Stress[1-0] | 5.4535372 | 15.9114 | 0.34 | 0.7377 | -29.21443 | 40.121508 |
| Stress[2-1] | -27.98656 | 28.28291 | -0.99 | 0.3419 | -89.60972 | 33.636602 |
| Stress[3-2] | 39.691817 | 35.40944 | 1.12 | 0.2842 | -37.45873 | 116.84236 |
| Stress[4-3] | -15.81006 | 22.14753 | -0.71 | 0.4890 | -64.06539 | 32.445272 |

PSQI = Pittsburgh Sleep Quality Index.

**Suboccipital Right**

**Table E.3**. Summary of significant associations between SD of the suboccipital right and BPSL-characteristics.

| **Term** | **Estimate** | **Standard Error** | **t Ratio** | **Prob>\|t\|** | **Lower 95%** | **Upper 95%** |
| --- | --- | --- | --- | --- | --- | --- |
| Intercept | 43.014113 | 34.63624 | 1.24 | 0.2606 | -41.73772 | 127.76594 |
| PSQI | -1.639591 | 1.982999 | -0.83 | 0.4400 | -6.491814 | 3.2126327 |
| Sedentary time | -5.059961 | 2.326473 | -2.17 | 0.0726 | -10.75264 | 0.6327145 |
| Anxiety[1-0] | -5.271046 | 12.38137 | -0.43 | 0.6852 | -35.56717 | 25.025081 |
| Anxiety[2-1] | -4.475373 | 22.19203 | -0.20 | 0.8468 | -58.77731 | 49.826561 |
| Anxiety[3-2] | 15.712656 | 26.04291 | 0.60 | 0.5684 | -48.01204 | 79.437353 |
| Anxiety[4-3] | -26.42653 | 16.35568 | -1.62 | 0.1573 | -66.44744 | 13.594381 |
| Stress[1-0] | -9.221366 | 11.48966 | -0.80 | 0.4528 | -37.33556 | 18.892824 |
| Stress[2-1] | -19.62195 | 20.59112 | -0.95 | 0.3774 | -70.0066 | 30.762698 |
| Stress[3-2] | 35.185278 | 26.34351 | 1.34 | 0.2301 | -29.27498 | 99.645537 |
| Stress[4-3] | 0.1574433 | 16.79855 | 0.01 | 0.9928 | -40.94714 | 41.262022 |
| HIT-6 | 0.1234862 | 0.59783 | 0.21 | 0.8432 | -1.339351 | 1.5863234 |

HIT = Headache Impact Test, PSQI = Pittsburgh Sleep Quality Index.

**Erector spine left**

**Table E.4**. Summary of significant associations between SD of the erector spine left and BPSL-characteristics.

| **Term** | **Estimate** | **Standard Error** | **t Ratio** | **Prob>\|t\|** | **Lower 95%** | **Upper 95%** |
| --- | --- | --- | --- | --- | --- | --- |
| Intercept | 110.40224 | 45.83559 | 2.41 | 0.0527 | -1.753405 | 222.55788 |
| PSQI | 3.662558 | 2.624185 | 1.40 | 0.2123 | -2.758592 | 10.083708 |
| Sedentary time | -0.456906 | 3.07872 | -0.15 | 0.8869 | -7.990262 | 7.0764492 |
| Anxiety[1-0] | -23.82519 | 16.38479 | -1.45 | 0.1961 | -63.91732 | 16.266946 |
| Anxiety[2-1] | 99.68448 | 29.36764 | 3.39 | **0.0146*** | 27.824457 | 171.5445 |
| Anxiety[3-2] | -85.25 | 34.46367 | -2.47 | **0.0482*** | -169.5796 | -0.920441 |
| Anxiety[4-3] | 6.1346553 | 21.64416 | 0.28 | 0.7864 | -46.82669 | 59.096001 |
| Stress[1-0] | -1.726785 | 15.20475 | -0.11 | 0.9133 | -38.93147 | 35.4779 |
| Stress[2-1] | -86.72381 | 27.24909 | -3.18 | **0.0190*** | -153.3999 | -20.0477 |
| Stress[3-2] | 74.934421 | 34.86148 | 2.15 | 0.0752 | -10.36854 | 160.23738 |
| Stress[4-3] | 10.879914 | 22.23023 | 0.49 | 0.6419 | -43.5155 | 65.275323 |
| HIT-6 | -1.591399 | 0.791133 | -2.01 | 0.0910 | -3.527233 | 0.3444349 |

HIT = Headache Impact Test, PSQI = Pittsburgh Sleep Quality Index; *= no significance was observed in the best model fit (below); Bold numbers = significance (p < .05).

**Table E.5.** Details on the associations between anxiety and SD of the erector spine left.

| **Source** | **Nparm** | **DF** | **Sum of Squares** | **F Ratio** | **Prob > F** |
| --- | --- | --- | --- | --- | --- |
| Anxiety | 4 | 4 | 1217.3968 | 0.5620 | 0.6944 |

**Erector spine right**

**Table E.6**. Summary of significant associations between SD of the erector spine right and BPSL-characteristics.

| **Term** | **Estimate** | **Standard Error** | **t Ratio** | **Prob>\|t\|** | **Lower 95%** | **Upper 95%** |
| --- | --- | --- | --- | --- | --- | --- |
| Intercept | 88.326065 | 74.83349 | 1.18 | 0.2825 | -94.7849 | 271.43703 |
| PSQI | -0.987073 | 4.284377 | -0.23 | 0.8254 | -11.47057 | 9.4964211 |
| Sedentary time | -0.94569 | 5.026473 | -0.19 | 0.8570 | -13.24503 | 11.353646 |
| Anxiety[1-0] | -9.424575 | 26.75063 | -0.35 | 0.7366 | -74.88101 | 56.031862 |
| Anxiety[2-1] | 19.320959 | 47.94709 | 0.40 | 0.7009 | -98.00134 | 136.64326 |
| Anxiety[3-2] | 55.63398 | 56.26712 | 0.99 | 0.3610 | -82.04671 | 193.31467 |
| Anxiety[4-3] | -51.86607 | 35.33734 | -1.47 | 0.1925 | -138.3334 | 34.601299 |
| Stress[1-0] | 9.0345089 | 24.82404 | 0.36 | 0.7284 | -51.70774 | 69.776754 |
| Stress[2-1] | -28.70451 | 44.48823 | -0.65 | 0.5427 | -137.5633 | 80.15428 |
| Stress[3-2] | 7.549875 | 56.9166 | 0.13 | 0.8988 | -131.72 | 146.81979 |
| Stress[4-3] | 15.794175 | 36.29419 | 0.44 | 0.6787 | -73.01451 | 104.60286 |
| HIT-6 | -0.927121 | 1.291644 | -0.72 | 0.4999 | -4.08766 | 2.2334191 |

HIT = Headache Impact Test, PSQI = Pittsburgh Sleep Quality Index.

**Tibialis anterior left**

**Table E.7**. Summary of significant associations between SD of the tibialis anterior left and BPSL-characteristics.

| **Term** | **Estimate** | **Standard Error** | **t Ratio** | **Prob>\|t\|** | **Lower 95%** | **Upper 95%** |
| --- | --- | --- | --- | --- | --- | --- |
| Intercept | 67.112492 | 62.6364 | 1.07 | 0.3252 | -86.15325 | 220.37823 |
| PSQI | -2.560247 | 3.586068 | -0.71 | 0.5021 | -11.33504 | 6.2145437 |
| Sedentary time | 1.31135 | 4.207209 | 0.31 | 0.7658 | -8.98332 | 11.60602 |
| Anxiety[1-0] | 6.6930596 | 22.39055 | 0.30 | 0.7751 | -48.09465 | 61.480764 |
| Anxiety[2-1] | -41.73836 | 40.1322 | -1.04 | 0.3384 | -139.9383 | 56.461595 |
| Anxiety[3-2] | 53.291651 | 47.09616 | 1.13 | 0.3010 | -61.94849 | 168.53179 |
| Anxiety[4-3] | -19.30706 | 29.57772 | -0.65 | 0.5381 | -91.68112 | 53.067008 |
| Stress[1-0] | 10.078177 | 20.77798 | 0.49 | 0.6448 | -40.7637 | 60.920056 |
| Stress[2-1] | 20.975029 | 37.23711 | 0.56 | 0.5937 | -70.14089 | 112.09094 |
| Stress[3-2] | -15.04151 | 47.63978 | -0.32 | 0.7629 | -131.6118 | 101.52883 |
| Stress[4-3] | -11.23198 | 30.37861 | -0.37 | 0.7243 | -85.56576 | 63.101793 |
| HIT-6 | -0.706256 | 1.081119 | -0.65 | 0.5378 | -3.35166 | 1.939148 |

HIT = Headache Impact Test, PSQI = Pittsburgh Sleep Quality Index.

**Tibialis anterior right**

**Table E.8**. Summary of significant associations between SD of the tibialis anterior right and BPSL-characteristics.

| **Term** | **Estimate** | **Standard Error** | **t Ratio** | **Prob>\|t\|** | **Lower 95%** | **Upper 95%** |
| --- | --- | --- | --- | --- | --- | --- |
| Intercept | 227.31904 | 103.673 | 2.19 | 0.0708 | -26.35956 | 480.99764 |
| PSQI | -2.838319 | 5.935499 | -0.48 | 0.6494 | -17.36196 | 11.685322 |
| Sedentary time | -16.27007 | 6.963584 | -2.34 | 0.0581 | -33.30935 | 0.7692046 |
| Anxiety[1-0] | -38.97042 | 37.05984 | -1.05 | 0.3335 | -129.6526 | 51.711734 |
| Anxiety[2-1] | 51.453245 | 66.42502 | 0.77 | 0.4680 | -111.0829 | 213.98942 |
| Anxiety[3-2] | -16.95536 | 77.95145 | -0.22 | 0.8350 | -207.6957 | 173.78497 |
| Anxiety[4-3] | -37.81192 | 48.95571 | -0.77 | 0.4692 | -157.6022 | 81.97839 |
| Stress[1-0] | -40.2273 | 34.39078 | -1.17 | 0.2865 | -124.3785 | 43.923899 |
| Stress[2-1] | -56.6586 | 61.63319 | -0.92 | 0.3934 | -207.4696 | 94.15239 |
| Stress[3-2] | 96.457006 | 78.85123 | 1.22 | 0.2671 | -96.485 | 289.39901 |
| Stress[4-3] | 32.36839 | 50.28131 | 0.64 | 0.5435 | -90.66555 | 155.40233 |
| HIT-6 | -1.779468 | 1.789421 | -0.99 | 0.3584 | -6.158023 | 2.5990863 |

HIT = Headache Impact Test, PSQI = Pittsburgh Sleep Quality Index.

**Appendix F – Detailed summary of the headache characteristics**

The table below provides information on the characteristics of cervicogenic headache. Data were retrieved from 18 patients through anamneses and a four-week headache diary [6].

**Table F.1.** Headache characteristics of patients with cervicogenic headache (n = 18).

| Characteristics | Results | | p |  |
| --- | --- | --- | --- | --- |
| Headache duration, mean hours/episode (SD) [CI] | 4.1 (1.6) [3.3;4.9] | | N/A |  |
| General headache intensity, mean VAS during a month (SD) [CI]  Left sided headache (n = 1), mean VAS during a month (SD) [CI]  Right sided headache (n = 17), mean VAS during a month (SD) [CI]  Peak headache intensity, mean VAS/episode during a month (SD) [CI]  Instantaneous headache-intensity, mean NPRS (SD) [CI] | 36 (21) [26;47]  30 (0) [30;30]  37 (22) [26;47]  61 (14) [54;67]  0.7 (1) [0.3;1.2] | | N/A |  |
| Headache-frequency, median days/month [IQR] | 11 [10;15.8] | | N/A |  |
| Headache location, n (%)  Ophthalmic, right  Frontal, right  Ophthalmic, left  Temporal, right  Frontal  Occipital | 12 (66.7), OR 4  2 (11.1)  1 (5.6)  1 (5.6)  1 (5.6)  1 (5.6) | | **.04^1^** |  |
| Referred pain from the neck, n (%)  Yes | N/A  18 (100) | | |  |
| Headache provocation (ADL), n (%)  Posture during desktop/laptop-work  Sitting, non-specified  Lying supine  Stress  Ironing  Posture during microscope-work | 10 (55.6), OR 4  2 (11.1)  2 (11.1)  2 (11.1)  1 (5.6)  1 (5.6) | | **.04^1^** |  |
| Headache provocation (PAIVM, upper-cervical uPA)  Yes, n (%) (Right/Left)  Mean NPRS (SD) [CI] | 18 (100) (17/1)  5.2 (2) [4.3;6.1] |  | N/A | |

n = number participants; CI = 95% level Confidence Interval; N/A = Not Applicable; ADL = Activities of Daily Living; NPRS = 11-point Numeric Pain Rating Scale; VAS = 100 mm Visual Analogue Scale, average VAS deducted from a four-week headache-diary; PAIVM = Passive Accessory Intervertebral Movement; uPA = unilateral Posterior-Anterior; IQR = 25-75% Interquartile Range; ^1^ = Fisher’s exact test; OR = Odds Ratio; Bold = p < .05.

**Appendix G – Raw data**

Table G.1 provides a summary of the raw data concerning the PPT-measurements.

**Table G.1.** Summary of the raw PPT-measurements.

Table G.2 provides a summary of the raw data concerning the Headache-Impact Test-6 (HIT-6).

**Table G.2.** Summary of the raw HIT-6 measurements.

Table G.3 provides a summary of the raw data concerning the Pittsburgh Sleep Quality Index (PSQI).

**Table G.3.** Summary of the raw PSQI measurements.

Table G.4 provides a summary of the raw data concerning the Depression, Anxiety and Stress Scale-21 (DASS-21).

**Table G.4.** Summary of the raw DASS-21 measurements.

Table G.5 provides a summary of the raw data concerning the sedentary -time.

**Table G.5.** Summary of the raw sedentary-time measurements

**References**

1. Lovibond, P.F. & Lovibond, S.H. The structure of negative emotional states: comparison of the Depression Anxiety Stress Scales (DASS) with the Beck Depression and Anxiety Inventories. *Behav Res Ther.* **33**, 335–343 (1995).

2. Martin, M., Blaisdell, B., Kwong, J.W. & Bjorner, J.B. The Short-Form Headache Impact Test (HIT-6) was psychometrically equivalent in nine languages. *J Clin Epidemiol.* ***57***, 1271–1278 (2004).

3. Kawata, A.K. *et al.* Psychometric properties of the HIT-6 among patients in a headache-specialty practice. *Headache.* ***45***, 638–643 (2005).

4. Mollayeva, T. *et al.* The Pittsburgh sleep quality index as a screening tool for sleep dysfunction in clinical and non-clinical samples: A systematic review and meta-analysis. *Sleep Med Rev.* ***25***, 52–73 (2016).

5. Mingels, S., Dankaerts, W., van Etten, L., Bruckers, L. & Granitzer, M. Spinal postural variability relates to biopsychosocial variables in patients with cervicogenic headache. *Sci Rep.* **11**, 13783 (2021).

6. Belgian Headache Society. *Hoofdpijndagboek* <http://www.belgianheadachesociety.be/bhs/download/headache-calendar-NL.pdf> (2022).
